# The Bishop's Last

**Published:** 1916-05-27

**Authors:** M. L. Johnson

**Affiliations:** Clifton, Bristol.


*To the Editor of* THE HOSPITAL.

### The Bishop's Last.

*To the Editor of* THE HOSPITAL.

SIR,—The association of the "last" and the "soul" professions has even higher sanction than that of the Bishop who told the talkative gentleman of the road, who demanded to know what line he travelled in, that "he travelled in souls." One of the Popes was the son of a shoemaker, and, so far from being ashamed of his parentage, had lasts in his escutcheon. The three Bishops' zeal in urging all citizens to forgo meat for one day a week recalls an incident described by the late Miss F. P. Cobbe in her racy autobiography. When a girl at a ladies' school in Brighton, for two years at which her bills amounted to £1,000, one Ash Wednesday the pupils were provided with a dish of salt fish. When it was removed to make room for the roast mutton one of the schoolmistresses addressed them, setting forth the merits of fasting. She insisted that they left the young ladies free to take meat or not, as they pleased. The mistresses, however, hoped that their pupils would fast, for "it would be good for their souls and their figures."

Another of Miss Cobbe's points was that "it was not surprising men were superior to women, considering they had so much better food and so much more sleep." Men believed in grateful appreciation of the gifts of Providence by their legitimate enjoyment, and that such use is necessary to the development of what is best in mankind.—Yours faithfully,

Clifton, Bristol. M. L. JOHNSON.

